# Bridging Gaps: Promoting Scientific Research in AOCMF Asia Pacific and Comparison with Latin America

**DOI:** 10.3390/cmtr18030035

**Published:** 2025-08-22

**Authors:** Radhika Menon, Takahiro Kanno, Yiu Yan Leung, Yeshaswini Thelekkat, Gopal Krishnan Kulandaswamy

**Affiliations:** 1 Department of Oral and Maxillofacial Surgery, Shimane University Faculty of Medicine, Izumo 693-0021, Japan; raadheekamenon@gmail.com; 2 Division of Oral and Maxillofacial Surgery, The University of Hong Kong, Hong Kong, China; mikeyyleung@hku.hk; 3 Department of Oral and Maxillofacial Surgery, PMS College of Dental Science and Research, Trivandrum 695028, India; yeshas26575@yahoo.com; 4 VMS Dental College, Vinayaka Mission Deemed University, Salem 636308, India; gops15@gmail.com

**Keywords:** craniomaxillofacial surgery, research barriers, mentorship, AO foundation, Asia Pacific region

## Abstract

Conducting scientific research in craniomaxillofacial surgery presents distinct challenges, particularly in the Asia Pacific region. This study aimed to assess research interests, barriers, and support needs among surgeons in the region through an anonymous online survey conducted via Google Forms from 12 to 31 May 2025, with 169 responses collected. The survey included 13 structured questions and an open-ended comment section. Findings were compared with a similar survey done in Latin America in 2024, to identify regional differences. The results revealed a significant gap in research participation, with 18.3% of Asia Pacific respondents having no publications, unlike Latin America, where all had at least one. Familiarity and participation in the *Arbeitsgemeinschaft für Osteosynthesefragen* Program for Education and Excellence in Research (AO PEER) were lower in Asia Pacific (29% and 6.5%), and greater challenges were reported in establishing topics, research methodology, and data collection. Although interest was high, only 42% conducted research frequently, and 90.5% indicated a need for mentorship. Despite higher awareness of AO grant opportunities (58%), barriers, like inadequate support for scientific research, lack of training, and limited time, persist. These findings highlight the need for AO Craniomaxillofacial surgery (AOCMF) to implement targeted strategies, such as research training, mentorship, promotion of funding opportunities, and support for multi-center collaborations, to enhance research participation across the region.

## 1. Introduction

Scientific research is the cornerstone of progress in craniomaxillofacial surgery, driving innovation and improving clinical outcomes. It not only contributes to medical progress but also plays a key role in a surgeon’s professional growth by enhancing analytical reasoning, communication skills, and the ability to evaluate new skills that directly benefit clinical practice [1]. Translational research helps bridge basic science and clinical application, enabling rapid implementation of innovations into healthcare systems, which are important for resource constrained regions seeking efficient healthcare improvements [2]. The need to build and encourage scientific growth in the Asia Pacific region comes with both opportunities and challenges due to its vast geographic, economic, and cultural diversity alongside wide differences in language, healthcare systems, economic status, and access to research facilities. While some nations have well-developed academic networks, others, especially low- and middle-income countries, still face problems, such as lack of funding, lack of awareness and research support [3], heavy clinical workloads with insufficient time for clinicians to engage in scientific work [4], minimal exposure to scientific writing during medical/dental education due to disparities in medical/dental education systems [1], and shortage of mentors to guide young surgeons and clinicians. Language also remains a huge barrier in the Asia Pacific region, with hundreds of native languages and dialects, like Mandarin, Hindi, Japanese, Korean, Thai, Maori, Bahasa, etc. English, not being the primary language, can make it difficult to access English-based scientific literature [5].

AOCMF stands out for its comprehensive approach to education, clinical training, and research support for surgeons worldwide. It is a nonprofit organization that promotes evidence-based practice, collaboration, and global networking, supported by an affordable CHF 100/year membership with no conflicts of interest. It offers structured maxillofacial trauma courses, hands-on osteosynthesis training, and access to innovative tools, such as the AO Surgery Reference and AO Video library. Each year, AOCMF supports around 85 sponsored clinical fellowships worldwide, ranging from 6 to 8 weeks, and facilitates faculty development programs to help practicing surgeons become educators. It also provides research grants to support innovative scientific projects, invests in clinical and pre-clinical research through its Research and Development and Clinical Investigation Divisions and publishes two specialty journals; *Craniomaxillofacial Trauma & Reconstruction (CMTR)* and *CMTR Open* [6]. Notably, the AO Program for Education and Excellence in Research (AO PEER) equips clinicians with the resources to design and publish clinical studies [7]. Despite its wide-ranging offerings, AOCMF remains underutilized in parts of the Asia Pacific region, highlighting the need to understand and address the barriers that limit research engagement among surgeons. This survey aimed to explore those challenges and identify opportunities to strengthen research participation in the Asia Pacific region through AOCMF.

Although a similar survey has been conducted in Latin America [8], it is not scientifically reliable to generalize those findings across different regions. The challenges faced by craniomaxillofacial surgeons can vary significantly depending on regional contexts. Therefore, understanding region-specific barriers is essential for designing effective and relevant interventions. This study was conducted in the Asia Pacific region with the aim of identifying the unique challenges faced by surgeons in engaging with scientific research, so that targeted strategies can be developed to support their needs and enhance research participation at a global level.

## 2. Materials and Methods

An online cross-sectional survey was conducted using Google Forms (Google LLC, Mountain View, CA, USA) and was distributed exclusively to active AOCMF members and craniomaxillofacial surgeons who had previously accessed AOCMF courses or educational events, using the email addresses available in the AO database. The survey was open from 12 to 31 May 2025. Participation was voluntary and anonymous. The questionnaire consisted of 13 multiple choice questions, similar to the one used in the Latin America survey, along with an optional comment section where respondents could share their experiences, suggestions, or challenges related to research. The estimated completion time was 5–10 min. To ensure data integrity and avoid duplicate entries, the survey was designed to allow only a single response per participant. All questions were mandatory, and each multiple choice question allowed only a single answer to maintain consistency and reduce response ambiguity. The full list of questions is provided in Table 1. A total of 169 responses were received. Responses were automatically recorded within the Google Forms platform and stored in Google Sheets, where its charting features were used to generate question-wise graphs and response percentages.

### Data Analysis

This preliminary exploratory survey did not include a formal priori sample size calculation, as the population size of eligible participants was unknown. A convenience sampling approach was used, following the methodology of Pereira et al. (2025) [8], and 169 responses were obtained from 18 countries. At a 95% confidence level and *p* = 0.5, the achieved sample of 169 responses corresponds to a margin of error of approximately ±7.5%.

## 3. Results

**Figure 1:**
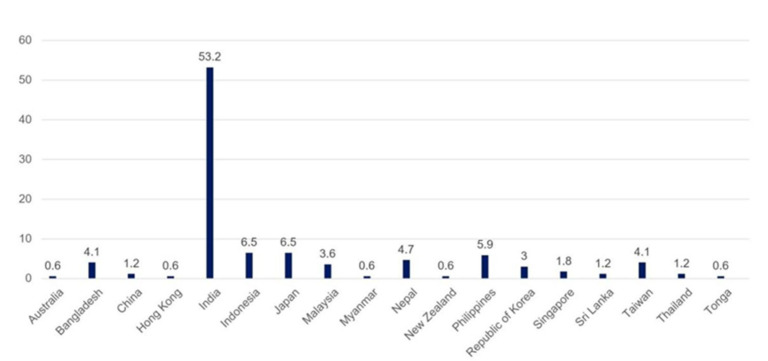
Graphic demonstrating the number of responses per country (%).

**Figure 2:**
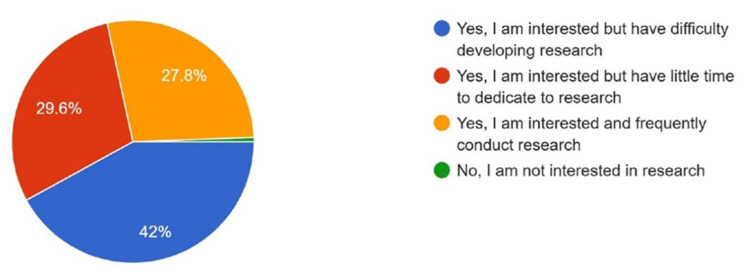
Graphic demonstrating the interest of the members in developing research.

A cross-sectional online survey was conducted using Google Forms between 12 and 31 May 2025. A total of 169 responses were obtained from participants across 18 countries, with the highest response received from India (Figure 1). The survey comprised 13 structured questions designed to assess the current state of research development in the region, as well as participants’ knowledge and awareness of the topic. Regarding research interest, 42% reported being interested but had difficulty developing research. About 29.6% were interested but had little time, while 27.8% actively conducted research with a small portion expressed no interest (Figure 2).

**Figure 3:**
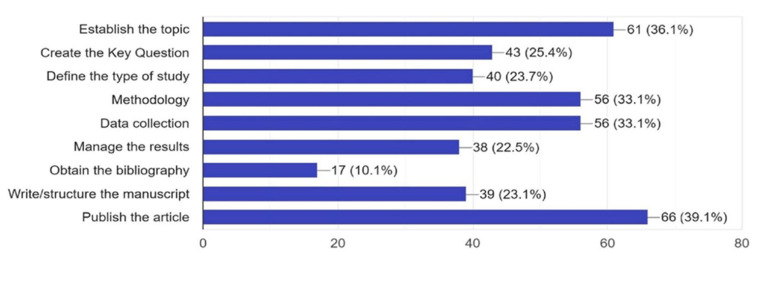
Graphic demonstrating the main difficulties of the members in developing research.

The main difficulties cited were Publishing the article (39.1%), Establishing the topic (36.1%), Methodology and Data collection (33.1%), Creating the key question (25.4%), Defining the type of study (23.7%), and Writing/structuring the manuscript (23.1%). Additionally, 22.5% had difficulty in Managing the results and 10.1% had issues Obtaining the bibliography (Figure 3).

**Figure 4:**
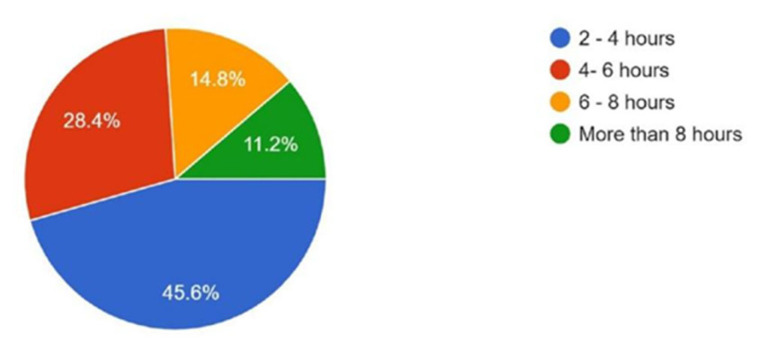
Graphic demonstrating the availability in time of the members to conduct research.

Regarding time availability for research, 45.6% reported having 2–4 h per week, 28.4% reported 4–6 h, 14.8% reported 6–8 h, and 11.2% reported more than 8 h per week (Figure 4).

**Figure 5:**
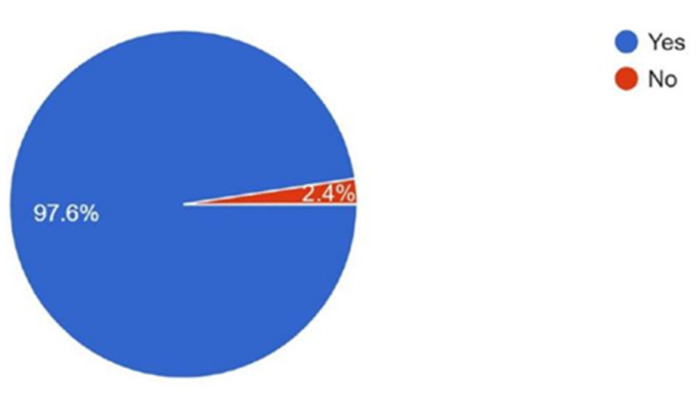
Graphic demonstrating the interest of the members in writing scientific articles.

When asked about their interest in writing scientific articles, 97.6% expressed interest, while only 2.4% reported no interest (Figure 5)**.**

**Figure 6:**
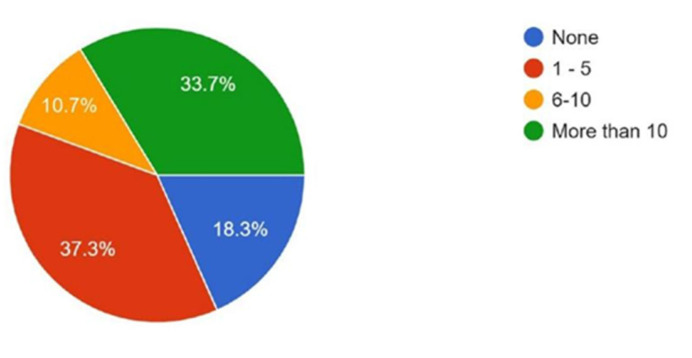
Graphic demonstrating the number of scientific articles published by the members.

**Figure 7:**
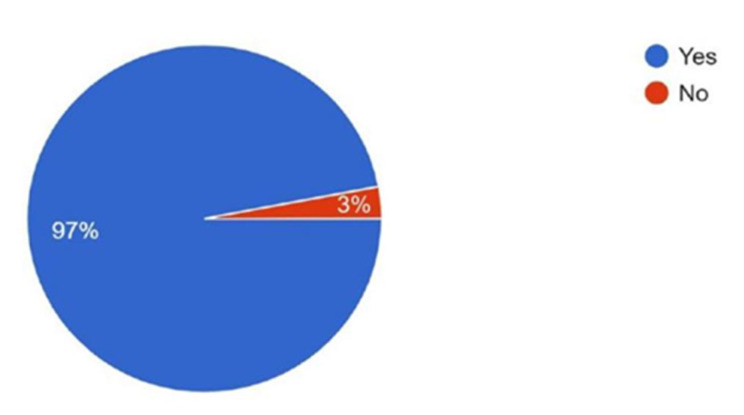
Graphic demonstrating the interest of the members in participating in a multi-center research study with AOCMF Asia Pacific.

Regarding publication history, 37.3% reported having fewer than five articles, 33.7% had more than ten publications, 10.7% had between five and ten, and 18.3% had no published articles (Figure 6). When asked about interest in participating in a multi-center research study with AOCMF Asia Pacific, 97% expressed interest, while 3% reported no interest (Figure 7). These data show that participants have some experience in article development but limited time dedication.

**Figure 8:**
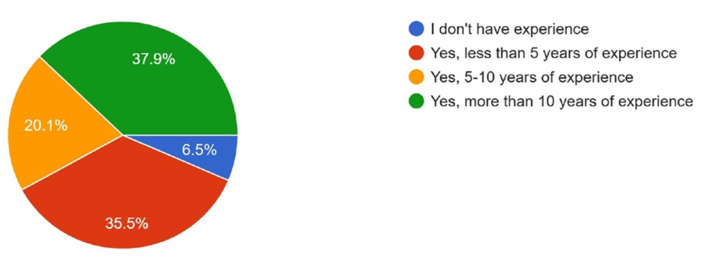
Graphic demonstrating the experience of members in craniomaxillofacial surgery.

Regarding experience in craniomaxillofacial surgery, 37.9% had more than 10 years of experience, 35.5% had less than 5 years, 20.1% had 5–10 years, and 6.5% reported having no experience in craniomaxillofacial surgery (Figure 8).

**Figure 9:**
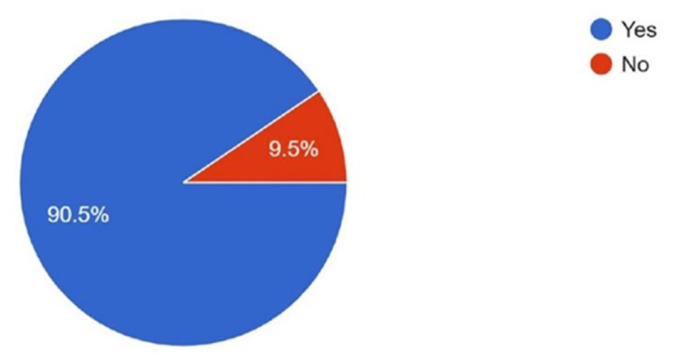
Graphic demonstrating the interest of members in obtaining mentorship for developing research projects.

**Figure 10:**
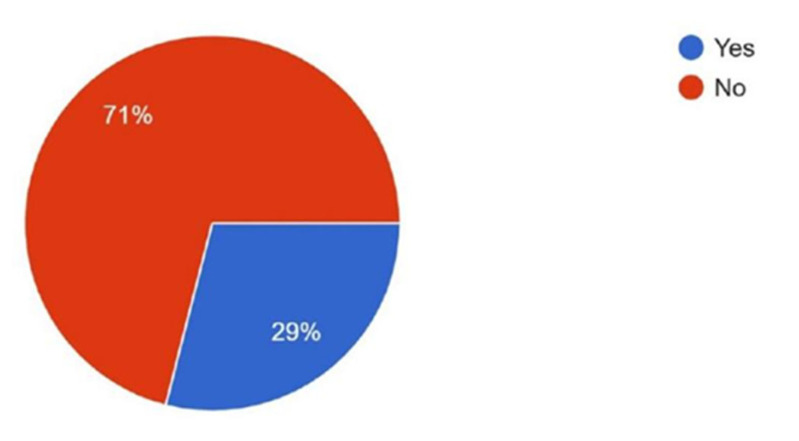
Graphic demonstrating the familiarity of members with the AO PEER programs.

When asked about interest in obtaining mentorship for developing research projects, 90.5% expressed interest, while 9.5% reported no interest (Figure 9). Familiarity with the AO PEER program was reported by 71% of respondents, while 29% were not familiar with it (Figure 10).

**Figure 11:**
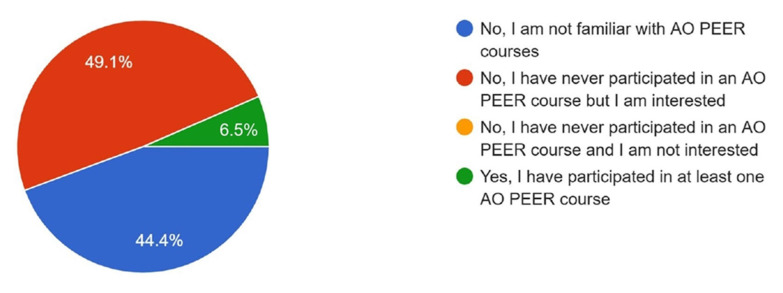
Graphic demonstrating the participation of members in the AO PEER programs.

**Figure 12:**
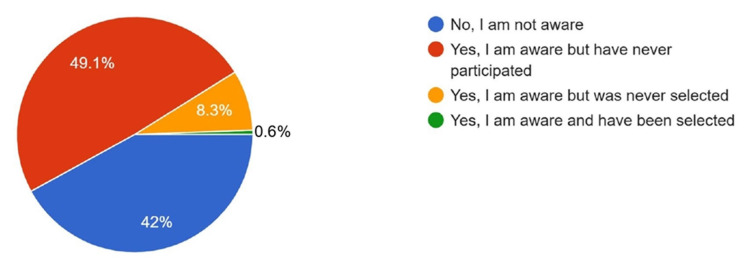
Graphic demonstrating the awareness of members about the research grant opportunities offered by the AO foundation.

When asked about participation in AO PEER courses, 49.1% reported that they had not participated but were interested, 44.4% were not familiar with the courses, and 6.5% had participated in at least one AO PEER course (Figure 11). Regarding awareness of research grant opportunities offered by the AO Foundation, 49.1% reported being aware but had never applied, 42% were not aware of these opportunities, 8.3% were aware but had not been selected, and only 0.6% reported being aware and successfully receiving a grant (Figure 12).

**Figure 13:**
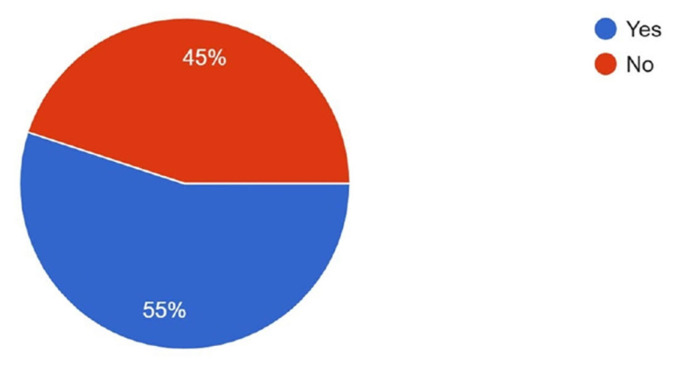
Graphic demonstrating the AO membership status of the respondents.

The survey was distributed to both active AOCMF members and surgeons who had previously attended AOCMF educational events. As a result, 55% of respondents reported being current AO members, while 45% were not (Figure 13).

The participants in the survey were also asked to provide comments related to the same topic, highlighting the challenges they face in research. A major concern was the lack of research training and mentorship, with many participants, including experienced surgeons, expressing difficulty in starting research due to limited guidance, lack of formal training in research methodology, and poor understanding of scientific writing. Many participants expressed difficulty in research execution, citing challenges in defining research questions, establishing methodologies, collecting sufficient clinical data, and preparing manuscripts with scientific writing standards to meet journal requirements for publication. Another significant challenge was limited funding and infrastructure, poor access to research facilities, and lack of institutional support, especially for those working in private clinics or resource-constrained settings. Additionally, there was low awareness of AO research resources, including the AO PEER program, research grants, and fellowship opportunities, pointing towards gaps in communication and outreach.

## 4. Comparative Analysis: Asia Pacific vs. Latin America

To assess regional variations, the results of the present survey were compared with those obtained from a similar study conducted in Latin America [8]. Key differences between the two regions are summarized in Table 2.

Overall, interest in research was high in both regions, with a slightly greater proportion of Asia Pacific respondents (99.4%) indicating interest compared to Latin America (96.5%). A lower percentage of Asia Pacific participants reported no interest in research (0.6%), suggesting a marginally higher level of motivation in the region.

Notable disparities were observed in research barriers and difficulties. Respondents from Asia Pacific reported significantly greater challenges in several foundational aspects of research, including establishing a topic (36.1% vs. 9.3%), formulating key research questions (25.4% vs. 2.3%), and collecting data (33.1% vs. 14%). Difficulties with research methodology, study design, and result management were also more pronounced in Asia Pacific. However, fewer respondents in Asia Pacific cited difficulties in structuring manuscripts compared to Latin America (23.1% vs. 32.6%).

While time availability for research was comparable between the two regions, a slightly higher proportion of Asia Pacific surgeons reported availability of more than 6 h per week. Despite this, Asia Pacific respondents had fewer publications overall, with 18.3% reporting no published work, a stark contrast to 0% in Latin America.

Interest in participating in multi-center research studies was high in both groups (97% vs. 95.3%). However, familiarity with and participation in the AO PEER program were significantly lower in Asia Pacific (familiarity: 29% vs. 65.1%; participation: 6.5% vs. 17.4%).

Interestingly, awareness of AO Foundation research grants was higher in the Asia Pacific cohort (58%) compared to Latin America (34.4%).

## 5. Discussion

Research plays a vital role in the advancement of clinical practice for craniomaxillofacial surgeons, enabling evidence based decision making, innovation, and improved patient outcomes. A surgeon should not be limited to the operating room but should also embrace the role of a scientist. The term “surgeon–scientist” typically refers to a surgeon actively engaged in bench research, often translational in nature, spanning a wide range of scientific fields. An aspiring surgeon scientist must be trained not only in the art of surgery but also in the principles and methods of scientific research. This dual role improves clinical practice, contributes to better patient outcomes, and supports the professional growth of the surgeon at a global level [9]. Despite its importance, active research participation among surgeons remains limited in many regions. To explore this issue within the Asia Pacific region, this survey was conducted and the findings reveal a strong research interest among Asia Pacific surgeons but significant obstacles in execution.

A similar survey was conducted in Latin America in 2024 [8]. This survey was carried out in the Asia Pacific region to identify regional differences and to address the specific needs and challenges faced by clinicians in the Asia Pacific. Responses were received from 13 countries in Latin America (86 responses) and 18 countries in the Asia Pacific region (169 responses), indicating slightly wider regional engagement in Asia Pacific. In Asia Pacific, research interest was notably high (99.4%), with fewer participants reporting no interest compared to Latin America (0.6% vs. 3.5%) with a greater proportion of respondents reporting difficulty in developing research (42% vs. 33.7%).

An exploratory study by Liu et al. (2021) [10] on clinical research training needs in Chinese hospitals reported challenges faced by clinicians, including difficulties in research design, topic development, and data handling, highlighting the need for structured clinical research training programs. These findings align with the results of this survey, as Asia Pacific respondents most commonly reported challenges with publishing articles (39.1%), establishing the topic (36.1%), and data collection (33.1%), all higher than in Latin America. Methodological issues (33.1% vs. 22.1%), managing results (22.5% vs. 14%), defining the type of study (23.7% vs. 18.6%), and creating the key question (25.4% vs. 2.3%) were also more prevalent in Asia Pacific. This indicates that targeted research training is essential for young researchers and clinicians, which can be addressed through structured mentorship programs, instructive lectures, and seminars, particularly those that guide surgeons on how to formulate and plan a research question, design a study, and write a scientific paper, and improved access to research guidelines and resources.

Asian clinicians often face heavy clinical workloads, which limits time for research activities [4]. Although Asia Pacific respondents reported slightly more available hours overall, especially in the 6–8 h range, most still dedicate limited time to research. This highlights the need to encourage clinicians to engage in research and to raise awareness of its importance for professional development and academic growth [2].

A cross-sectional descriptive study by Okoduwa et al. (2018) [11] identified major barriers to research and publishing, such as lack of mentorship, inadequate training, and limited understanding of the publication process. Notably, 57% of respondents had never published in a peer-reviewed journal, attributing this to long review timelines, lack of departmental motivation, high rejection rates, and publication fees. Similarly, in this survey, although the majority of Asia Pacific respondents had published articles, 18.3% had not published a single article, in contrast to Latin America, where all participants had at least one publication. This highlights a significant gap in research participation and underscores the need for AOCMF to offer structured guidance on scientific writing, covering journal selection, understanding the peer review process, identifying indexed journals, and adhering to standard scientific research writing guidelines, to improve acceptance rates and publication success.

Familiarity with the AO PEER program was significantly low in Asia Pacific (29%) compared to Latin America (65.1%) and participation in AO PEER courses was also low in Asia Pacific (6.5%) compared to Latin America (17.4%), though nearly half of Asia Pacific respondents (49.1%) had never participated but expressed interest. This indicates that many surgeons are still unaware of AO PEER programs. Despite strong interest, low awareness prevents them from taking full advantage of available resources. Therefore, AO must strengthen awareness efforts through focused communication, educational campaigns, and integration of AO PEER information into existing events and courses, enabling more clinicians to benefit from these programs and enhance their research skills.

A recent online survey conducted in Asia by Tong et al. (2025) [3] identified lack of funding, awareness, and organizational support as key barriers in conducting research. Similarly, a qualitative study by Ichsan et al. (2018) [12] in Indonesia highlighted a lack of funding and awareness as a major barrier to primary care research participation in lower- and middle-income countries. The results of this survey reflect similar challenges, with awareness of AO research grant opportunities higher in Asia Pacific (58%) compared to Latin America (34.4%), yet nearly half of Asia Pacific respondents aware of the grants had never participated. Even with strong interest, increased awareness of research grants and support is essential for boosting research participation in the region.

Asia Pacific represents an ideal region for advancing clinical research, given its large patient population and the evident interest in scientific research among surgeons. With access to diverse clinical cases, the key lies in systematically collecting patient data and guiding it towards scientific writing. Publishing such work not only benefits the global surgical community by sharing new ideas and techniques, but also enhances the professional development, recognition, and international collaboration opportunities for the contributing surgeons. The findings of this study provide valuable feedback to AOCMF, highlighting the specific barriers and needs of clinicians in the region, and can guide the development of focused initiatives to support surgeons in becoming active contributors to scientific research and evolving into surgeon–scientists.

## 6. Limitations

The limitations of this survey include its cross-sectional design, potential response bias due to voluntary participation and selection bias due to the inclusion of only AOCMF members or prior participants in AO activities, which may not represent all craniomaxillofacial surgeons in the Asia Pacific region. As this was a preliminary survey, the focus was limited to craniomaxillofacial surgeons from the broader head and neck surgical team. This allowed for targeted analysis, but future studies, including plastic and reconstructive surgeons, as well as regional and national level studies are needed to enhance the applicability of the findings and provide deeper insights into the challenges and barriers faced in research activities across different countries.

## 7. Conclusions

Encouraging surgeons to engage in scientific research is essential for advancing craniomaxillofacial care in the Asia Pacific region. Emphasizing the importance of translational research, increasing awareness of available programs and grant opportunities, providing structured research training programs, instructive lectures, and workshops on the basics of scientific writing, and fostering multi-center collaborative studies can significantly enhance research participation. The barriers preventing surgeons from engaging in research have now been clearly identified, providing AOCMF with the necessary insight to develop targeted strategies. With this understanding, AOCMF is well-positioned to lead these efforts and empower clinicians to grow as surgeon–scientists.

## Figures and Tables

**Table 1:** Survey questions with response options.

Question Number	Question	Response Options
1	Are you interested in conducting research?	Yes/No
2	What are the main difficulties you face in conducting research?	Multiple selections: Funding/Time/Training/Mentorship/Infrastructure/Other
3	What is your availability to dedicate to a research project (in weekly hours)?	0–2 h/2–5 h/5–10 h/>10 h
4	Are you interested in writing scientific articles?	Yes/No
5	How many scientific articles have you published?	None/1–5/6–10/>10
6	Are you interested in participating in a multi-center research study with AOCMF Asia Pacific?	Yes/No/Maybe
7	Do you have experience in CMF surgery?	Yes/No
8	Are you interested in obtaining mentorship for developing your research project?	Yes/No
9	Are you familiar with the AO PEER program?	Yes/No
10	Have you participated in any AO PEER course(s)?	Yes/No
11	Are you aware of the research grant opportunities offered by the AO Foundation?	Yes/No
12	Please indicate your location/country from the list below	Drop-down list of Asia Pacific countries
13	Are you currently an AO Member?	Yes/No/Not Sure

**Table 2:** Comparison of research metrics: Asia Pacific vs. Latin America.

Category	Asia Pacific (%)	Latin America (%)	Notable Difference
***Research Interest***			
Interested in Research	99.4	96.5	Asia Pacific slightly higher
Not Interested in Research	0.6	3.5	Asia Pacific more motivated
***Barriers***			
Difficulty Developing Research	42	33.7	Higher in Asia Pacific
Little Time for Research	29.6	34.9	Slightly higher in Latin America
Frequently Conduct Research	27.8	27.9	Almost similar
***Main Research Difficulties***			
Establishing a Topic	36.1	9.3	Significantly challenging in Asia Pacific
Creating Key Question	25.4	2.3	Significantly challenging in Asia Pacific
Defining the Type of Study	23.7	18.6	Asia Pacific struggles more
Research Methodology	33.1	22.1	Asia Pacific finds it difficult
Collecting Data	33.1	14	Asia Pacific finds it difficult
Managing the Results	22.5	18.6	Asia Pacific finds it difficult
Obtaining the Bibliography	10.1	11.6	Almost similar
Writing/Structuring the Manuscript	23.1	32.6	Less challenging in Asia Pacific
Publishing the Article	39.1	30.2	More challenging in Asia Pacific
***Time Availability***			
<4 h/week	45.6	48.8	Comparable
4 h/week	28.4	32.6	Comparable
8 h/week	14.8	8.1	More in Asia Pacific
>8 h/week	11.2	10.5	Almost similar
***Publication History***			
<5 Publications	37.3	41.9	Less publications by Asia Pacific
Up to 10 Publications	10.7	22.1	Less publications by Asia Pacific
>10 Publications	33.7	36	Less publications by Asia Pacific
No Publications	18.3	0	Significant gap in Asia Pacific
***Interest in a Multi-center Research Study***			
Yes	97	95.3	High interest in both the regions
***AO PEER Program***			
Familiar	29	65.1	Significantly lower familiarity in Asia Pacific
Participation in AO PEER Courses	6.5	17.4	Significantly less participation by Asia Pacific members
***Research Grants by the AO Foundation***			
Aware	58	34.4	More awareness in Asia Pacific

## Data Availability

No new data were created or analyzed in this study. Data sharing is not applicable to this article.
